# A Vision/Inertial Navigation/Global Navigation Satellite Integrated System for Relative and Absolute Localization in Land Vehicles

**DOI:** 10.3390/s24103079

**Published:** 2024-05-12

**Authors:** Yao Zhang, Liang Chu, Yabin Mao, Xintong Yu, Jiawei Wang, Chong Guo

**Affiliations:** 1 National Key Laboratory of Automotive Chassis Integration and Bionics, Jilin University, Changchun 130025, China; zyao18@mails.jlu.edu.cn (Y.Z.); chuliang@jlu.edu.cn (L.C.); maoyb21@mails.jlu.edu.cn (Y.M.); wjw20@mails.jlu.edu.cn (J.W.); 2 China FAW Group Co., Ltd., Changchun 130000, China; yuxintong@faw.com.cn; 3 Changsha Automobile Innovation Research Institute, Changsha 410005, China

**Keywords:** state estimator, vehicle localization, sensor fusion, adaptive mechanism

## Abstract

This paper presents an enhanced ground vehicle localization method designed to address the challenges associated with state estimation for autonomous vehicles operating in diverse environments. The focus is specifically on the precise localization of position and orientation in both local and global coordinate systems. The proposed approach integrates local estimates generated by existing visual–inertial odometry (VIO) methods into global position information obtained from the Global Navigation Satellite System (GNSS). This integration is achieved through optimizing fusion in a pose graph, ensuring precise local estimation and drift-free global position estimation. Considering the inherent complexities in autonomous driving scenarios, such as the potential failures of a visual–inertial navigation system (VINS) and restrictions on GNSS signals in urban canyons, leading to disruptions in localization outcomes, we introduce an adaptive fusion mechanism. This mechanism allows seamless switching between three modes: utilizing only VINS, using only GNSS, and normal fusion. The effectiveness of the proposed algorithm is demonstrated through rigorous testing in the Carla simulation environment and challenging UrbanNav scenarios. The evaluation includes both qualitative and quantitative analyses, revealing that the method exhibits robustness and accuracy.

## 1. Introduction

To address the inherent limitations in individual sensors, researchers have shifted their focus towards the development of multi-sensor fusion localization systems. These systems capitalize on the strengths of diverse sensors to augment the precision and robustness of the localization system. Consequently, there is a growing emphasis on the application of sensor fusion methods to ascertain vehicle positions. Currently, simultaneous localization and mapping (SLAM) stands as the prevailing solution for autonomous vehicle localization, incorporating the fusion of GNSS/RTK, laser radar, LIDAR-based prior maps, and IMU-based high-quality positioning [1,2]. However, the widespread adoption of laser radar sensors in autonomous vehicles is impeded by their high cost.

In contrast, visual odometry (VO) and visual simultaneous localization and mapping (SLAM) have garnered significant attention due to their advantages, including low cost, compact size, and straightforward hardware configuration. Nevertheless, the purely visual approach lacks robustness in the presence of sparse-textured areas, motion blur, sharp turns, and lighting variations. Therefore, the inertial measurement unit (IMU) is integrated to provide short-term motion constraints and the absolute scale of motion. Systems tightly coupling visual observations and IMU measurements are denoted as visual–inertial navigation systems (VINSs), proficient in estimating the six-degree-of-freedom (DOF) pose of a vehicle. Even in conditions of rapid motion or substantial changes in lighting, this system consistently achieves high precision and robust localization results [3,4,5,6,7].

The most direct approach to fuse visual and inertial measurements is through a filter-based loosely coupled framework [8,9]. However, this straightforward strategy overlooks the correlations between different sensor data, leading to suboptimal localization accuracy. Consequently, tightly coupled fusion methods have been introduced, wherein the tight coupling of visual and inertial measurements falls into filter-based and optimization-based categories. Filter-based tightly coupled fusion methods, such as [3,6,10], concurrently optimize the states of the camera and IMU. These methods typically restrict the number of landmarks, preserving only the most recently detected features in the state vector to ensure a manageable problem complexity. Nevertheless, they commonly encounter a shared challenge: visual–inertial navigation systems (VINSs) constitute nonlinear systems, necessitating the linearization of nonlinear measurements before processing, which may introduce significant errors [11]. In contrast, optimization-based methods convert sensor fusion into a graph-based nonlinear least squares problem, delivering superior accuracy compared to filter-based methods, with the drawback of increased computational time. Consequently, effective IMU pre-integration techniques are widely employed in optimization-based tightly coupled visual–inertial odometry (VIO) methods [4,5,12], as well as in [7,13,14], where manifolds are used instead of Euler angles to parameterize the rotation group for enhanced computational efficiency. Optimization-based methods typically optimize the most recent states within a limited-size sliding window while marginalizing past states and measurements [5]. Ref. [5] stands out as one of the most popular open-source VIO systems, exhibiting an excellent performance on the KITTI dataset. However, depending solely on local relative pose estimation is insufficient with autonomous vehicles, prompting the need for an absolute localization method to map local state estimates into a global coordinate system.

Given that the Global Navigation Satellite System (GNSS) offers absolute positioning information in the Earth coordinate system, a natural approach involves integrating local positioning results into the absolute position data from GNSS for precise global localization. Notably, ref. [15] devised a tightly coupled visual–inertial odometry (VIO) system augmented by intermittent GNSS measurements, yielding consistent global localization results. This approach concurrently addresses spatiotemporal sensor calibration and state initialization. Additionally, ref. [16] introduced an innovative filter-based estimator, amalgamating GNSS measurements with visual–inertial data. It concurrently estimates the extrinsic rotation between GNSS and VIO results online, achieving robust global localization. Beyond filter-based methods, ref. [17] proposed a sliding window optimization-based strategy that positions the vehicle in the global coordinate system by fusing long-range stereo vision, inertial integration, and limited GNSS information. Ref. [18] introduced a tightly coupled framework that integrates visual–inertial odometry with global positioning measurements. Recent advancements, as demonstrated by methods [19,20], tightly couple visual–inertial SLAM with raw GNSS measurements, yielding globally consistent localization information. However, tightly coupled methods present challenges in both complex initialization and limited scalability. Integrating new sensors necessitates algorithm redesign, making system expansion difficult. Ref. [21] presents a loosely coupled sensor fusion framework capable of attaining locally accurate and globally drift-free attitude estimation, showcasing a commendable performance in practical scenarios. However, it is noteworthy that this system does not explicitly initialize the coordinate transformation from East North Up (ENU) to visual–inertial odometry (VIO).

Additionally, it overlooks the potential issues that may arise from the failure of a sensor.

This study introduces the integration of Global Navigation Satellite System (GNSS) signals to enhance the performance of visual–inertial navigation systems (VINSs) for achieving locally accurate and globally drift-free attitude estimation. The proposed approach is easy to extend and involves a two-stage initialization method to acquire initial poses at both local and global levels. Additionally, an adaptive fusion mechanism is introduced to ensure effective global pose estimation even in the event of VIO or GNSS failure. Algorithm validation is conducted in simulation environments and challenging urban road scenarios. The primary contributions of this work include the following:

- Two-Stage Initialization Method: The introduction of a two-stage initialization method leveraging inertial, camera, and asynchronous GPS measurement data. This method facilitates a coarse estimation of visual–inertial odometry (VIO) initialization parameters and the transformation matrix between the VIO coordinate system and the global East North Up (ENU) system when the vehicle is stationary. Further optimization of relevant initialization parameters occurs as the vehicle is in motion.
- Adaptive Fusion Mechanism: The implementation of an adaptive fusion mechanism that incorporates anomaly detection for VIO and GPS information. In the event of VIO or GPS failure, this mechanism enables the system to continue estimating the global pose of the vehicle, ensuring robust and continuous vehicle localization.
- Algorithm Validation in Simulations and Challenging Urban Roads: The validation of the algorithm’s effectiveness in both simulation environments and challenging urban roads. The validation process includes qualitative and quantitative analyses. Through these experiments, the study demonstrates the satisfactory performance of the proposed method across different scenarios, highlighting its robustness and accuracy.

## 2. System Overview

**Figure 1:**
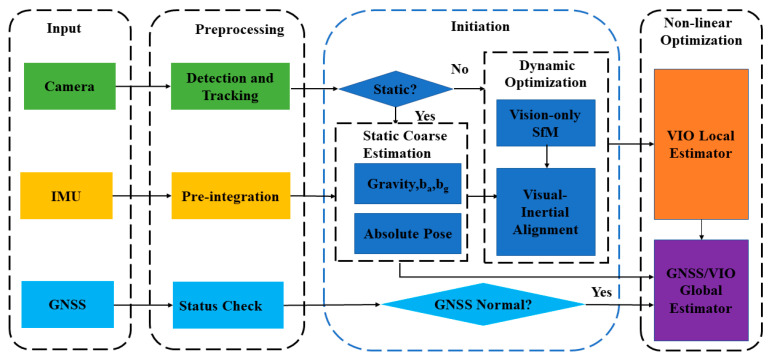
The scheme of proposed system.

The structure of our proposed system is illustrated in Figure 1.

The system integrates inputs such as images, inertial measurements, and GNSS data. The high-frequency IMU between the two images will be pre-integrated. The GNSS frequency is low and unstable. When new GNSS data are obtained, the positioning status is detected first, and the positioning anomaly and some GNSS data far away from the image acquisition time will be discarded to avoid major positioning errors. At the initial moment when the vehicle is stationary, IMU data are utilized to estimate rough accelerometer and gyroscope biases, the direction of gravity, and the coordinate transformation from ENU to VIO. The transition from a static to dynamic state is identified by analyzing disparities between consecutive images. Upon detecting vehicle motion, the initialization parameters initially estimated during the stationary phase are optimized to conclude the VIO initialization. Following initialization, the VIO local estimator generates local pose estimates. These local pose estimates, in conjunction with GNSS information, serve as inputs to the global estimator. A nonlinear optimization process is then employed to produce the final six-degree-of-freedom (DOF) global pose results.

## 3. Methods

**Figure 2:**
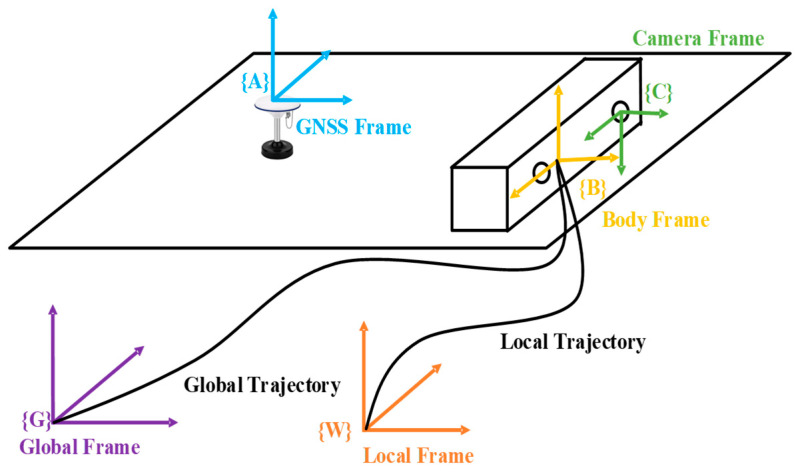
Diagram of the coordinates involved.

This section begins with the definitions of the coordinate systems. The involved coordinates in this paper are shown in Figure 2, including the sensor frames: the camera frame {C}, the IMU frame {B} (vehicle frame), and the GNSS frame {A}. The phase center of the GNSS antenna serves as the coordinate origin for {A}. {W} is the reference frame for local odometry, aligning the gravity vector with the z-axis, and the origin is set at the vehicle’s starting point, $g^{W}={\left[0,0,9.81\right]}^{T}$. {G} represents the global East-North-Up (ENU) frame, serving as the reference frame for global poses, with an origin identical to the local reference frame {W}. The following symbols will be used. We define ${\left(•\right)}^{G}$ and ${\left(\cdot \right)}^{W}$ to represent matrices (or vectors) in {G} and {W}, respectively. ${\left(\cdot \right)}^{C_{k}}$, ${\left(\cdot \right)}^{B_{k}}$, and ${\left(\cdot \right)}^{A_{k}}$ are the camera, IMU, and GNSS data frames when capturing the kth image. $R_{A}^{B}$ is considered the rotation matrix from frame {A} to {B}, with the corresponding Hamilton quaternion form denoted as $q_{A}^{B}$. $p_{A}^{B}$ and $v_{A}^{B}$ represent the position and velocity vectors of {A} in {B}, and $T_{A}^{B}$ is the homogeneous expression of the transformation matrix from {A} to {B}. External parameters between sensors are employed to transform measurement data from different frames to a unified coordinate system. The external parameters for Camera-IMU include $R_{C}^{B}$ and $p_{C}^{B}$. $L_{A}^{B}$ represents the GNSS-IMU external parameter, known as the lever arm, and all involved external parameters are calibrated offline. ${\left\lfloor \cdot \right\rfloor }_{\times }$ denotes vector cross-product, ⊗ denotes quaternion multiplication, and finally, $\left(\hat{•}\right)$ is used to represent measurement data containing noise.

### 3.1. System Preliminaries

#### 3.1.1. IMU Measurements and Pre-Integration Theory

IMU sensors typically consist of a 3-axis gyroscope and a 3-axis accelerometer, allowing for the measurement of angular velocity and acceleration of the inertial sensor (i.e., body frame) with respect to the inertial frame. IMU measurements combine the force for countering gravity and the platform dynamics, subject to acceleration bias, gyroscope bias, and additional noise. The raw measurements from the gyroscope and accelerometer $\hat{\omega }$ and $\hat{a}$ are given by Equation (1):

(1) $$\begin{array}{c} {\hat{a}}_{t}=a_{t}+b_{a_{t}}+R_{W}^{t}g^{W}+n_{a}\\ {\hat{\omega }}_{t}=\omega_{t}+b_{w_{t}}+n_{w} \end{array}$$

Assuming that the additional noise in the accelerometer and gyroscope measurements are Gaussian white noise, $n_{a}∼N(0,\sigma_{a}^{2})$, $n_{w}∼N(0,\sigma_{w}^{2})$. The accelerometer bias and gyroscope bias are modeled as a random walk by Equation (2), with their derivatives being Gaussian white noise, $n_{b_{a}}∼N(0,\sigma_{b_{a}}^{2})$, $n_{b_{w}}∼N(0,\sigma_{b_{w}}^{2})$.

(2) $${\dot{b}}_{a_{t}}=n_{b_{a}},\;{\dot{b}}_{w_{t}}=n_{b_{w}}.$$

To avoid recomputing integrals when linearization points change, we adhere to the approach outlined in [5]. Given the biases, we compute the relative motion increment between two consecutive keyframes using Equation (3). Importantly, this increment remains independent of the attitude and velocity at time $t_{k}$.

(3) $$\begin{array}{l} \alpha_{B_{k+1}}^{B_{k}}=\iint_{t\in [t_{k},t_{k+1}]}R_{t}^{B_{k}}\left({\hat{a}}_{t}-b_{a_{t}}\right)dt^{2}\\ \beta_{B_{k+1}}^{B_{k}}=\underset{t\in [t_{k},t_{k+1}]}{\int }R_{t}^{B_{k}}\left({\hat{a}}_{t}-b_{a_{t}}\right)dt\\ \gamma_{B_{k+1}}^{B_{k}}=\underset{t\in [t_{k},t_{k+1}]}{\int }\frac{1}{2}\Omega ({\hat{\omega }}_{t}-b_{w_{t}})\gamma_{t}^{B_{k}}dt \end{array}$$

where Ω(·) is defined by Equation (4):

(4) $$\Omega \left(\omega \right)=\left[\begin{array}{cc} -{\left\lfloor \omega \right\rfloor }_{\times } & \omega \\ -\omega^{T} & 0 \end{array}\right],{\left\lfloor \omega \right\rfloor }_{\times }=\left[\begin{array}{ccc} 0 & -\omega_{z} & \omega_{y}\\ \omega_{z} & 0 & -\omega_{x}\\ -\omega_{y} & \omega_{x} & 0 \end{array}\right]$$

#### 3.1.2. Monocular Vision

Applying the monocular pinhole camera model, upon receiving a new image, features are detected using Harris corner detection [22]. Subsequently, the KLT sparse optical flow algorithm [23] is employed to track these newly detected features. The mapping relationship between landmark points and features is expressed through Equation (5):

(5) $$\pi \left(x,l\right)=K\left[\begin{array}{cc} R_{W}^{C} & p_{W}^{C} \end{array}\right]L^{W}$$

where K denotes the camera intrinsic calibration matrix. x represents the inverse of the camera pose, l represents the projection of a landmark in the three-dimensional world onto the camera plane, while $L^{W}$ denotes the three-dimensional position of the landmark in the local reference frame {W}.

#### 3.1.3. GNSS Measurements

Taking the first GNSS position measurement as the origin of the global reference frame {G}, the GNSS observations at time $t_{k}$ are represented by a general Equation (6):

(6) $$z_{k}^{A}=h{\left(x_{k}\right)}^{A}+n^{A}$$

where $h{\left(\cdot \right)}^{A}$ is the function connecting the IMU frame {B} and GNSS measurements, and $n^{A}$ represents the measurement noise. In fact, GNSS observations can be expressed using Equation (7):

(7) $$\begin{array}{l} p_{A_{k}}^{G}=p_{W}^{G}+R_{W}^{G}p_{A_{k}}^{W}\\ p_{A_{k}}^{W}=p_{B_{k}}^{W}+R_{B}^{W}L_{A}^{B} \end{array}$$

where $p_{A_{k}}^{G}$ is the position of the GNSS antenna phase center in the global reference frame {G} at time $t_{k}$.

#### 3.1.4. Nonlinear Optimization

Vehicle localization can be viewed as a state estimation problem, which can be transformed into a maximum likelihood estimation (MLE) problem. MLE is composed of the joint probability distribution of vehicle states over a certain period. Under the assumption that all measurements are independent, this problem is typically formulated as Equation (8):

(8) $$\chi^{*}=\underset{\chi }{\mathrm{argmax}}\prod_{n}^{t=0}\prod_{k\in S}p(z_{t}^{k}|\chi )$$

where S represents measurements from the camera, IMU, or other sensors. Assuming that sensor measurements follow a Gaussian distribution $p(z_{t}^{k}|\chi )∼N\left({\bar{z}}_{t}^{k},\Omega_{t}^{k}\right)$, the negative log likelihood of Equation (8) can be expressed as Equation (9):

(9) $$\begin{array}{c} \chi^{*}=\arg\max\prod_{n}^{t=0}\prod_{k\in S}exp(-\frac{1}{2}{\left\|z_{t}^{k}-h_{t}^{k}(\chi )\right\|}_{\Omega_{t}^{k}}^{2})\\ =\arg\underset{X}{\min}\sum_{n}^{t=0}\sum_{k\in S}{\left\|z_{t}^{k}-h_{t}^{k}(\chi )\right\|}_{\Omega_{t}^{k}}^{2} \end{array}$$

The Mahalanobis norm is defined as $∥r{∥}_{\Omega }^{2}=r^{T}\Omega^{-1}r$, and the sensor model h(·) is defined by Equations (1), (5) and (6). The state estimation is then transformed into an iteratively optimized nonlinear least squares problem, where vertices represent the variables to be optimized and edges denote error terms. A graph corresponding to any nonlinear least squares problem can be constructed.

### 3.2. Initiation

To achieve an improved localization performance, initializing the system is essential. A two-stage initialization method is proposed to fully exploit measurement data from the vehicle’s startup to the commencement of motion. In the stationary phase, initialization involves estimating gravity direction, gyroscope biases, accelerometer biases, and a rough estimate of the absolute pose. As the vehicle begins to move, the parameters estimated during the stationary phase are rapidly optimized, and scale is restored, thus completing the initialization process.

#### 3.2.1. Rough Estimation of Static Parameters

Initially, using stationary state data to estimate IMU biases, during this phase, the vehicle’s initial velocity and position are both zero. The gravity acceleration measured in the {B} frame is obtained from Equation (10):

(10) $$Z_{0}=\sum_{i=1}^{m}\left({\hat{a}}_{k}\right)/\|\sum_{i=1}^{m}\left({\hat{a}}_{k}\right){\|}_{2}$$

where ${\hat{a}}_{k}$ is the observation of the kth IMU accelerometer; m is the total number of IMU observations obtained in the stationary state of the vehicle. If the parallax is less than the threshold, then the vehicle is considered to be stationary. The projection of the x-axis direction vector of the world frame in the IMU frame is obtained using Equation (11):

(11) $$X_{0}=e-(e^{T}\cdot Z_{0})\cdot Z_{0}$$

The projection of the y-axis direction vector of the world frame in the IMU frame is obtained using Equation (12):

(12) $$Y_{0}={\left\lfloor X_{0}\right\rfloor }_{\times }\;Z_{0}$$

Therefore, the rotation matrix from the VIO frame {W} to the IMU frame {B} is obtained using Equation (13):

(13) $$R_{W}^{B}=[X_{0}\;Y_{0}\;Z_{0}]$$

The biases of the accelerometer and gyroscope are calculated using Equation (14):

(14) $$\begin{array}{l} b_{a}=\frac{1}{m}\sum_{m}^{i=1}(a_{i})-R_{w}^{B}\cdot g^{W}\\ b_{g}=\frac{1}{m}\sum_{m}^{i=1}(\omega_{i}) \end{array}$$

Based on double-vector attitude determination [24], a rough estimate of $R_{W}^{G}$ is obtained, completing the static initialization phase.

#### 3.2.2. Dynamic Optimization

When there is stable feature tracking and sufficient disparity (exceeding 20 pixels) between the latest image and all the previously stored images in the sliding window, the method proposed in [5] is employed. Initially, a visual reconstruction is conducted, followed by visual–inertial alignment. The first camera frame, denoted as {C_0_}, is set as the reference. Subsequently, the rough estimate parameters obtained from Equations (10), (13), and (14) are utilized as initial values for optimization. The optimization process begins with the refinement of gyroscope biases, followed by the initialization of the velocity, gravity vector, and scale factor. In this process, accelerometer biases are simultaneously considered, defining the system state as Equation (15):

(15) $$\chi_{I}=\left[v_{B_{0}}^{B_{0}},v_{B_{1}}^{B_{1}},\ldots v_{B_{n}}^{B_{n}},g^{c_{0}},s,b_{a}\right]$$

where $v_{B_{k}}^{B_{k}}$ represents the velocity of {B} at the time of capturing the kth image, $g^{C_{0}}$ is the gravity vector in the reference frame, and s scales the normalized reconstruction to metric units. By solving the system described in Equation (16), the velocity of {B} within the window, the gravity vector in the visual reference frame {C_0_}, and the scale parameter are obtained.

(16) $$\underset{X_{I}}{\min}\sum_{k\in ℬ}\|{\hat{z}}_{B_{k+1}}^{B_{k}}-H_{B_{k+1}}^{B_{k}}\chi_{I}{\|}^{2}$$

After further adjustment of the gravity vector, $g^{C_{0}}$ is rotated to align with the z-axis in the {W} frame, resulting in the calculation of $R_{C_{0}}^{W}$. All variables are adjusted to the {W} frame to complete the initialization.

### 3.3. VIO Local Estimator

For local pose estimation, an existing visual–inertial odometry (VIO) algorithm is employed. There are many excellent open-source VIO algorithms available, and this paper utilizes the algorithm from [5]. In the sliding window, the algorithm estimates the poses of several IMU frames along with the depth of visual features. The state is defined as Equation (17):

(17) $$\begin{array}{c} \chi_{W}=[x_{0},x_{1},\cdots x_{n},\lambda_{0},\lambda_{1},\cdots \lambda_{m}]\\ x_{k}=\left[\begin{array}{c} p_{B_{k}}^{W},v_{B_{k}}^{W},q_{B_{k}}^{W},b_{a},b_{g} \end{array}\right],k\in [0,n], \end{array}$$

where the *k*th IMU state $x_{k}$ is composed of the position $p_{B_{k}}^{W}$, velocity $v_{B_{k}}^{W}$, orientation $q_{B_{k}}^{W}$, gyroscope bias $b_{g}$, and accelerometer bias $b_{a}$, representing the position of the IMU center relative to the local reference frame {W}. The orientation is represented using quaternion.

The first IMU pose serves as the reference frame. When a feature is first observed in the camera frame, it is parameterized using inverse depth λ. At this point, the state estimation can be represented as Equation (18):

(18) $$\begin{array}{c} \underset{X_{W}}{\min}\left\{∥r_{P}-H_{P}\chi {∥}^{2}+\sum_{k\in B}\|r_{B}({\hat{z}}_{B_{k+1}}^{B_{k}},\chi ){\|}_{P_{B_{k+1}}^{B_{k}}}^{2}+\;\sum_{(W,j)\in C}\rho (∥r_{C}({\hat{z}}_{W}^{c_{j}},\chi ){∥}_{P_{W}^{c_{j}}}^{2})\right\} \end{array}$$

where $r_{B}({\hat{z}}_{B_{k+1}}^{B_{k}},\chi )$ and $r_{C}({\hat{z}}_{W}^{c_{j}},\chi )$ represent the inertial and visual residuals, respectively. The prior $\left\{r_{P},H_{P}\right\}$ contains information about the marginalized state history. The term ρ(·) denotes a robust kernel function [25], which effectively suppresses the influence of outliers. For a detailed explanation, refer to [5]. The VIO local estimator achieves precise real-time 6-DoF local pose estimation.

### 3.4. GNSS/VIO Global Estimator

#### 3.4.1. Pose Graph Structure

**Figure 3:**
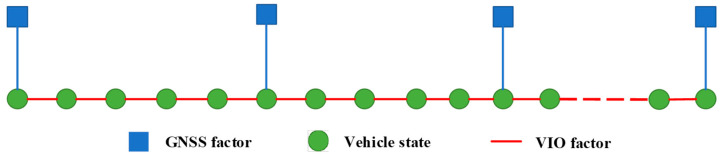
Pose graph structure.

The global pose estimation problem can be represented using the pose graph in Figure 3. Each VIO node represents the estimated local six-degree-of-freedom pose. Given the high accuracy of visual–inertial odometry in the short term, we introduce the relative pose between these two nodes as a constraint to the pose graph. In instances where a node is associated with GNSS measurements, GNSS constraints are also included as global constraints in the pose graph. This approach proves effective in mitigating the impact of cumulative errors, considering the non-accumulative nature of GNSS measurements.

#### 3.4.2. Adaptive Fusion Mechanism

Based on nonlinear optimization theory, the global state is defined as Equation (19):

(19) $$\begin{array}{c} \chi_{G}=[x_{0},x_{1},\cdots x_{n}]\\ x_{k}=\left[\begin{array}{c} p_{B_{k}}^{G},q_{B_{k}}^{G} \end{array}\right],k\in [0,n], \end{array}$$

where $p_{B_{k}}^{G}$ and $q_{B_{k}}^{G}$ are the position and orientation in global reference frame {G}, respectively.

Due to the intricacies of autonomous driving scenarios, there are instances where the local visual–inertial odometry (VIO) system may encounter tracking failures, leading to a restart, and sometimes GNSS signals may be limited. Therefore, an adaptive mechanism is employed. When the local VIO system encounters a failure, reliance is placed on GNSS signals. Similarly, when GNSS signals are restricted, local VIO measurements are utilized. Upon recovery of the local VIO system, visual–inertial odometry (VIO) factors are reintroduced into the pose graph, and when GNSS signals recover, global factors are reintroduced into the pose graph. Therefore, the adaptive fusion optimization problem for GNSS and VIO factors can be represented as Equation (20):

(20) $$\begin{array}{c} \chi^{*}=\arg\underset{\chi_{G}}{\min}\sum_{k\in B}\Phi {\left\|z_{B_{k}}^{W}-h_{B_{k}}^{W}(\chi )\right\|}_{\Omega_{B_{k}}^{W}}^{2}+\\ \Psi \cdot \sum_{k\in A}\rho \left({\left\|z_{A_{k}}^{G}-h_{A_{k}}^{G}(\chi )\right\|}_{\Omega_{A_{k}}^{G}}^{2}\right) \end{array}$$

where Φ is the VIO adaptive coefficient, with a value of 1 when VI is normal and a value of 0 otherwise. Similarly, Ψ is the GNSS adaptive coefficient, with a value of 1 in the normal state and a value of 0 otherwise.

The detection of local visual–inertial odometry (VIO) failure is executed by measuring the relative translation or rotation between two consecutive frames. If the change between these frames exceeds a predefined threshold, then VIO failure is considered. 

At this point, let Φ=0. If VIO is normal, then let Φ=1.

The anomaly detection of GNSS primarily relies on the position state and covariance matrix of its measurement information. The detection of abnormal states involves two steps. Firstly, the positioning state of GNSS must satisfy Equation (21); otherwise, GNSS is directly considered to be in an abnormal state.

(21) $$\begin{array}{l} status>0\\ \sigma_{RMS}=\sqrt{\frac{\sigma_{\lambda }^{2}+\sigma_{\phi }^{2}+\sigma_{h}^{2}}{3}}\leq {\bar{\sigma }}_{RMS} \end{array}$$

where status is the positioning state, with 0 indicating an invalid position. $\sigma_{RMS}$ is the covariance of the positioning results. ${\bar{\sigma }}_{RMS}$ is the positioning covariance threshold. $\sigma_{\lambda },\sigma_{\varphi },\sigma_{h}$ are the covariances of the longitude, latitude, and altitude measurement.

Secondly, utilizing the chi-squared detection method, GNSS state determination is carried out. The covariance of longitude, latitude, and elevation measurements is employed to calculate the information matrix of GNSS residuals. The covariance matrix and information matrix for the kth frame of GNSS measurements are given by Equation (22):

(22) $$\begin{array}{c} P_{A_{k}}=diag\left(\sqrt{\frac{\sigma_{\lambda }^{2}+\sigma_{\phi }^{2}}{2}},\sqrt{\frac{\sigma_{\lambda }^{2}+\sigma_{\phi }^{2}}{2}},\sigma_{h}^{2}\right),\\ \Sigma_{A_{k}}=P_{A_{k}}^{-1} \end{array}$$

When GNSS measurements follow a normal distribution, the corresponding GNSS residuals $r_{G_{k}}$ follow a Gaussian distribution with a mean of zero and a variance of $P_{A_{k}}$. Supposing that there are p frames of GNSS measurement data in the optimized sliding window, the length of the data window is p, and the dimension of the chi-squared test is 3, then all GNSS data in this data window follow a chi-squared distribution with 3p degrees of freedom. The abnormal detection function can be designed as Equation (23):

(23) $$E_{k}=\sum_{k\in A}r_{A_{k}}P_{A_{k}}^{-1}r_{A_{k}}^{T}$$

Choosing a 95% confidence level and based on 3p degrees of freedom, the threshold can be obtained from the table. When $E_{k}$ is less than the threshold, the GNSS is considered in a normal state. At this point, let Ψ=1; otherwise, let Ψ=0.

Local VI factors can come from any local state estimator. Considering two consecutive measurement frames, the visual–inertial factor is defined as Equation (24):

(24) $$\begin{array}{c} z_{B_{k}}^{W}-h_{B_{k}}^{W}(\chi )=z_{B_{k}}^{W}-h_{B_{k-1}}^{W}(x_{k-1},x_{k})\\ =[\begin{array}{c} \alpha q_{B_{k-1}}^{W}\left(p_{B_{k}}^{W}-p_{B_{k-1}}^{W}\right)-\alpha q_{B_{k-1}}^{G}\left(p_{B_{k}}^{G}-p_{B_{k-1}}^{G}\right)\\ \beta ({q_{B_{k-1}}^{W}}^{-1}q_{B_{k}}^{W})⊖({q_{B_{k-1}}^{G}}^{-1}q_{B_{k}}^{G}) \end{array}] \end{array}$$

where ⊖ represents quaternion subtraction and α,β are the weighting factors for translation and rotation, respectively.

By setting the first GNSS measurement as the origin, we can obtain GNSS measurements in the global ENU (East North Up) coordinate system. At this point, where the global reference frame {G} and local reference frame {W} origins coincide, the GNSS factor can be defined as Equation (25):

(25) $$\begin{array}{l} z_{A_{k}}^{G}-h_{A_{k}}^{G}(\chi )=z_{A_{k}}^{G}-h_{A_{k}}^{G}(x_{k})\\ ={\hat{p}}_{A_{k}}^{G}-(p_{B_{k}}^{G}+R_{B_{k}}^{G}L_{A}^{B}) \end{array}$$

At this stage, the system can discern whether various factors are anomalous. Ultimately, optimization and solving for the global pose are conducted using Ceres Solver [26].

## 4. Experiments and Results

The proposed positioning method is implemented within the Robot Operating System (ROS) framework. To assess the effectiveness of the proposed system, experiments were conducted in the simulation environment Carla [27] and on the publicly available UrbanNav dataset [28]. All the result analyses were conducted using the EVO [29].

### 4.1. Simulation

#### 4.1.1. Step

The simulation environment was in Town 5 within Carla, an open-source simulator designed for autonomous driving research, supporting flexible sensor configurations and environmental conditions. An RGB camera from Carla was employed with an image size of 800 × 600, a field of view (FOV) set to 90, and an image frequency of 20 Hz. A virtual IMU with a frequency of 100 Hz was utilized, with standard deviations associated with the white noise of the accelerometer and the gyroscope set to 0.01 m/s^2^ and 0.001 rad/s, respectively. The standard deviation of the gyroscope bias random walk was set to 1.0 × 10^−5^ rad/s. Additionally, a virtual GNSS sensor outputted longitude, latitude, and altitude information at 10 Hz, with the error set to 1 m. All sensors were rigidly mounted on the vehicle. The vehicle was set to autonomous mode, transitioning from a stationary position to motion.

#### 4.1.2. Results

In order to assess the practicality of the proposed initialization algorithm and adaptive mechanism, a comprehensive comparison was performed against VINS_FUSION. The initial step involved setting up an ideal environment within the Carla simulation framework to commence the initialization of the local visual–inertial odometry (VIO) system. Subsequently, the average scale error and initialization time were computed and compared with VINS-FUSION (without GNSS). Additionally, the feasibility and trajectory accuracy of the proposed adaptive algorithm were validated by comparing it to VINS-FUSION (with GNSS).

**Figure 4:**
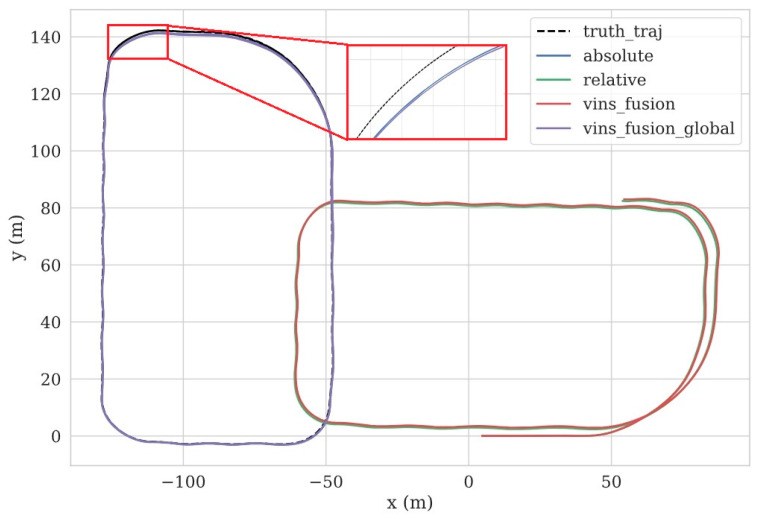
The trajectories in Carla. **Truth_traj** represents the ground truth trajectory, **absolute** is the trajectory obtained from the proposed adaptive algorithm, and **relative** is the trajectory obtained from the proposed two-stage initialization process. **Vins_fusion** is the trajectory obtained from VINS-FUSION (without GNSS). **Vins_fusion_global** is the trajectory obtained from VINS-FUSION (with GNSS).

Figure 4 illustrates the trajectory results for different methods in the Carla simulation. Our proposed initialization approach shows a high degree of precision comparable to VINS_FUSION. In scenarios where VIO failures are absent, our adaptive method exhibits a trajectory highly similar to VINS-FUSION. Additionally, there is a noticeable discrepancy between the global absolute trajectory and the local relative trajectory, primarily attributed to the four-degree-of-freedom non-observability in local pose estimation (i.e., x, y, z, and yaw). Cumulative drift leads to an imperfect overlap of trajectories when traversing the same path twice locally, while the global pose trajectory demonstrates good consistency.

Table 1 presents the root-mean-square error (RMSE) of absolute pose error (APE) for each trajectory, along with statistics on representative initialization parameters. The average parameters are computed by averaging the results from five trials. The results demonstrate that our two-stage initialization approach performs better than VINS-FUSION (without GNSS) in terms of scale estimation precision and RMSE reduction, achieved within a shorter initialization duration. Furthermore, our adaptive algorithm yields an RMSE of 0.578 m, indicating an enhanced localization accuracy when compared to VINS-FUSION (with GNSS).

### 4.2. Public Datasets

#### 4.2.1. Introduction of Datasets

The reliability of the proposed system was validated on the challenging UrbanNav dataset, specifically leveraging the UrbanNav-HK-Data20190314. The data collection platform comprises an INS/IMU (Xsens-Mti10, sourced by Xsens Technologies, Enschede, The Netherlands), multiple GNSS receivers (u-blox M8T), a 3D lidar sensor, and several monocular cameras. The ground truth is provided by the Novatel SPAN-CPT, an advanced RTK GNSS/INS integrated navigation system.

#### 4.2.2. Results

**Figure 5:**
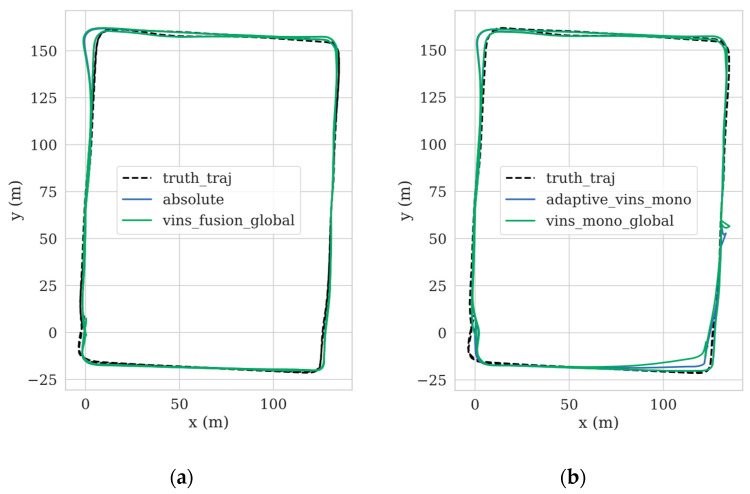
The trajectories in the UrbanNav-HK-Data20190314. (**a**) shows the result trajectories obtained from our proposed algorithm compared to VINS-FUSION (with GNSS), **absolute** is the trajectory obtained from the proposed adaptive algorithm, **and vins_fusion_global** is the trajectory obtained from the VINS-FUSION; (**b**) shows the result trajectories obtained when our method and VINS-FUSION were extended to VINS-MONO. **adaptive_vins_mono** is the trajectory obtained from the proposed adaptive algorithm, and **vins_mono_global** is the trajectory obtained from VINS-FUSION. The red box indicates visual–inertial odometry (VIO) failure.

**Figure 6:**
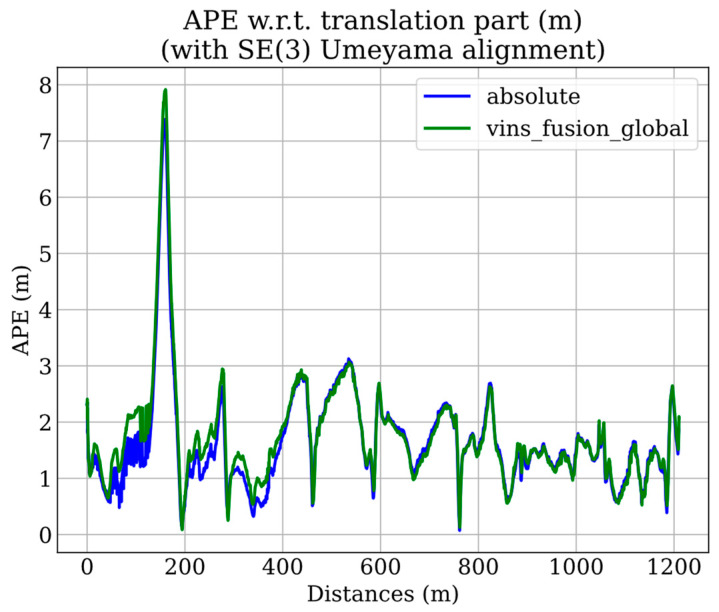
Different system trajectories’ APE compared to ground truth. The x-axis, “Distances”, represents the distance from the starting point in meters.

The efficacy of our adaptive algorithm was verified on the complex UrbanNav-HK-Data20190314 dataset, known for its dynamic obstacles in urban alleyways. A comparative analysis was performed against the advanced visual–inertial navigation system VINS-FUSION (with GNSS) to evaluate the algorithm’s performance. Additionally, to broaden the scope of our method, we extended the comparison to VINS-MONO. The trajectory outcomes are illustrated in Figure 5. In order to assess the overall accuracy of the localization trajectory, we present a comparison of the absolute pose error (APE) with VINS-FUSION in Figure 6.

Figure 5a depicts the comparison between our proposed method and VINS-FUSION (with GNSS). Under stable visual–inertial odometry (VIO) conditions, both methods exhibit similar levels of accuracy. However, the absolute position error (APE) analysis in Figure 6 reveals that our proposed algorithm achieves a lower APE compared to VINS-FUSION, indicating a superior performance. Figure 5b illustrates the trajectory outcomes of our proposed algorithm extended to VINS-MONO. Throughout the algorithm’s operation, two VIO system failures occurred due to rapid maneuvers and texture-deficient environments. In such instances, our proposed algorithm demonstrated a better trajectory alignment with ground truth, thereby offering higher precision.

In order to further evaluate the accuracy of localization trajectories, a quantitative analysis was conducted on the trajectory, with the results presented in Table 2. It indicates that the overall length of paths estimated using our proposed algorithm is more accurate compared to VINS-FUSION. Additionally, in cases where visual–inertial odometry (VIO) experiences failure, the RMSE of the APE for our proposed algorithm is 1.902 m, which is 5% lower than that of VINS-FUSION. This signifies a higher level of accuracy with our proposed algorithm.

## 5. Conclusions

This paper introduces a relative and absolute positioning system for ground vehicles. The entire system, starting from initialization, employs a two-stage initialization method to fully utilize measurement data from inertial sensors, cameras, and asynchronous GNSS. During vehicle stationary periods, it roughly estimates the initialization parameters of visual–inertial odometry (VIO) and the transformation matrix between the VIO coordinate system and ENU. When the vehicle is in motion, it further optimizes the relevant initialization parameters. Additionally, an adaptive fusion mechanism is utilized to ensure smooth system operation even when a single sensor signal is unavailable. Finally, experiments are conducted in both the Carla simulation environment and a dataset from complex real-world scenarios. The proposed system demonstrates robustness and accuracy. Since there is no hard synchronization in time between GNSS and other sensors, and the time offset between sensors will change with time, it is necessary to incorporate time-offset estimation into future work.

## Figures and Tables

**Table 1:** The root-mean-square error (RMSE) of the absolute pose error (APE) for each trajectory and result of representative initialization parameters.

	RMSE (m)	Average Initialization Time (ms)	Average Scale Error (%)
relative	2.272	83.423	1.14%
vins_fusion	2.462	93.541	1.32%
absolute	0.578	NA	NA
vins_fusion_global	0.645	NA	NA

**Table 2:** Dataset and trajectory quantitative analysis results.

	Time Duration (s)	Length (m)	VI Collapse Times	GNSS Available Rate ^1^ (%)	RMSE (m)
truth_traj	279.91	1209.45	NA	NA	NA
absolute	279.88	1217.13	0	81.40	1.902
vins_fusion_global	279.88	1217.79	0	100	2.014
adaptive_vins_mono	279.88	1218.31	2	81.40	2.997
vins_mono_global	279.88	1234.82	2	100	3.111

^1^ GNSS Available Rate refers to the ratio of valid GNSS signals after chi-square testing to the total received signals. A rate of 100% indicates direct usage of received GNSS signals without anomaly detection.

## Data Availability

The data that has been used is confidential.
